# A predictive model to explore risk factors for Henoch–Schönlein purpura nephritis in children: a retrospective cross-sectional study

**DOI:** 10.3389/fpubh.2025.1507408

**Published:** 2025-03-19

**Authors:** Qianwen Yang, Maoyang Zhang, Zilong Dong, Fang Deng

**Affiliations:** ^1^Department of Nephrology, Children’s Hospital of Anhui Medical University, Children’s Medical Center of Anhui Medical University, Hefei, Anhui, China; ^2^Department of Pediatrics, Hefei Maternal and Child Health Hospital, Hefei, Anhui, China

**Keywords:** Henoch-Schönlein purpura, nephritis, nomogram, children, predictive models

## Abstract

**Objective:**

The risk factors for Henoch–Schönlein purpura nephritis (HSPN) remain largely unclear, particularly in family environment and vaccination. This study aimed to develop a predictive framework to quantify the risk of HSPN by examining family environmental factors and COVID-19 vaccination outcomes in children with Henoch–Schönlein purpura (HSP) in Anhui, China.

**Methods:**

This study retrospectively analyzed 362 children diagnosed with HSP at Anhui Children’s Hospital between January 2020 and February 2024. A questionnaire was designed to collect information from enrolled children. For patients with incomplete medical records, parents were contacted via phone or the questionnaire was sent to them to complete the survey. After data collection, the patients were split randomly into a training group and a validation group at a 7:3 ratio, univariate and multivariate logistic regression analyses were performed to identify risk factors for nephritis, and a nomogram was constructed from these factors to provide a visual prediction of the likelihood of nephritis in HSP. The nomogram’s performance was evaluated in both the training and validation groups using the area under the receiver operating characteristic (AUC) curve, calibration plots, and decision curve analysis (DCA).

**Results:**

The study identified family income/month, age of onset, BMI, number of recurrences, and COVID-19 vaccination status as independent risk factors for HSPN. A nomogram was subsequently developed afterward using these factors. In the training group, the nomogram achieved an area under the curve (AUC) of 0.83 (95% CI: 0.78–0.88), while in the validation group, the AUC was 0.90 (95% CI: 0.84–0.96), demonstrating strong predictive performance. The calibration curve showed that the nomogram’s predictions were well-aligned with the actual outcomes. Additionally, DCA indicated that the nomogram provided considerable clinical net benefit.

**Conclusion:**

The nomogram offers accurate risk prediction for nephritis in children with HSP, helping healthcare professionals identify high-risk patients early and make informed clinical decisions.

## 1 Introduction

HSP is a systemic vasculitis that affects small blood vessels, primarily in children, involving skin, joints, gastrointestinal tract, and kidneys (1). Also referred to as IgA vasculitis (IgAV), the extent of kidney involvement is a key determinant of long-term outcomes. As many as 50% of children with IgAV develop kidney involvement as the disease progresses (2). While kidney symptoms are typically mild, some patients may progress to nephrotic syndrome or renal failure. Despite the generally favorable prognosis of IgAV, kidney damage remains the main cause of mortality and a critical factor in long-term outcomes (3). Therefore, early recognition of risk factors for kidney involvement in HSP is crucial for intervening effectively and managing the condition.

Although the exact etiology and pathogenesis of HSP remain unclear, particularly with respect to predicting kidney involvement or disease progression in children, it is understood that its onset may result from a combination of genetic susceptibility, environmental factors, and infections. The specific triggers are still being studied and may include infections, medications, and vaccinations (4–7). The most commonly identified trigger is a prior upper respiratory tract infection, although there have been reports suggesting that vaccinations may also induce HSP.

The COVID-19 pandemic, which began in December 2019, rapidly escalated into a global crisis within just a few months, with cases rising exponentially. Several studies have described an association between COVID-19 and vasculitis, particularly in children. In fact, several studies have highlighted an association between COVID-19 and vasculitis, particularly in children, with reports of HSP cases occurring after COVID-19 infection or vaccination. This suggests that a shared mechanism of immune activation might be involved (8, 9). Additionally, emerging evidence suggests a potential link between COVID-19 vaccination and kidney diseases, though data are still limited and require further exploration (10–12).

In addition to these biological triggers, the family environment plays a critical role in human health. It is well established that family socioeconomic status, family structure, home environment, parenting behaviors, parental mental health, and substance use significantly influence health outcomes (13). However, the complex interplay of these interconnected factors complicates the study of diseases like HSP. Confounding factors within the family environment can obscure the relationship between specific risk factors and HSP, making it more difficult to fully understand the disease’s underlying mechanisms. However, these factors often interact in complex ways, introducing confounding effects that can obscure the relationships between specific risk factors and diseases like HSP. To address these challenges, this study systematically examines the demographic, health, and medical profiles of children with HSP and their parents in Anhui, China, focusing on family environment and COVID-19 vaccination status. By doing so, it aims to identify novel risk factors for kidney involvement in HSP and provide insights into its prevention and management.

## 2 Materials and methods

### 2.1 Participants

A convenience sampling method was employed to select children under 14 years old with HSP who were admitted to Anhui Provincial Children’s Hospital between January 2020 and February 2024, as participants in this study. All procedures were conducted in compliance with the applicable guidelines and regulations. The diagnoses of IgAV and IgA vasculitis nephritis (IgAVN) were made based on the EULAR/PRINTO/PRES criteria (14). Firstly, the presence of non-thrombocytopenic purpura predominantly occurring on the lower limbs; secondly, the presence of at least one of the following four criteria: (i) abdominal pain, (ii) histological evidence of positive IgA deposition, (iii) arthritis or arthralgia, and (iv) renal involvement. Additionally, the criteria for IgA vasculitis with nephritis (IgAVN) in this study were met if either of the following was present: (i) histopathological confirmation from a renal biopsy, or (ii) 24-h urinary protein excretion ≥150 mg/day. Other kidney diseases (such as nephrotic syndrome or IgA nephropathy), autoimmune diseases, malignancies, or systemic vasculitis were excluded based on clinical diagnostic criteria and laboratory test results.

**Figure 1:**
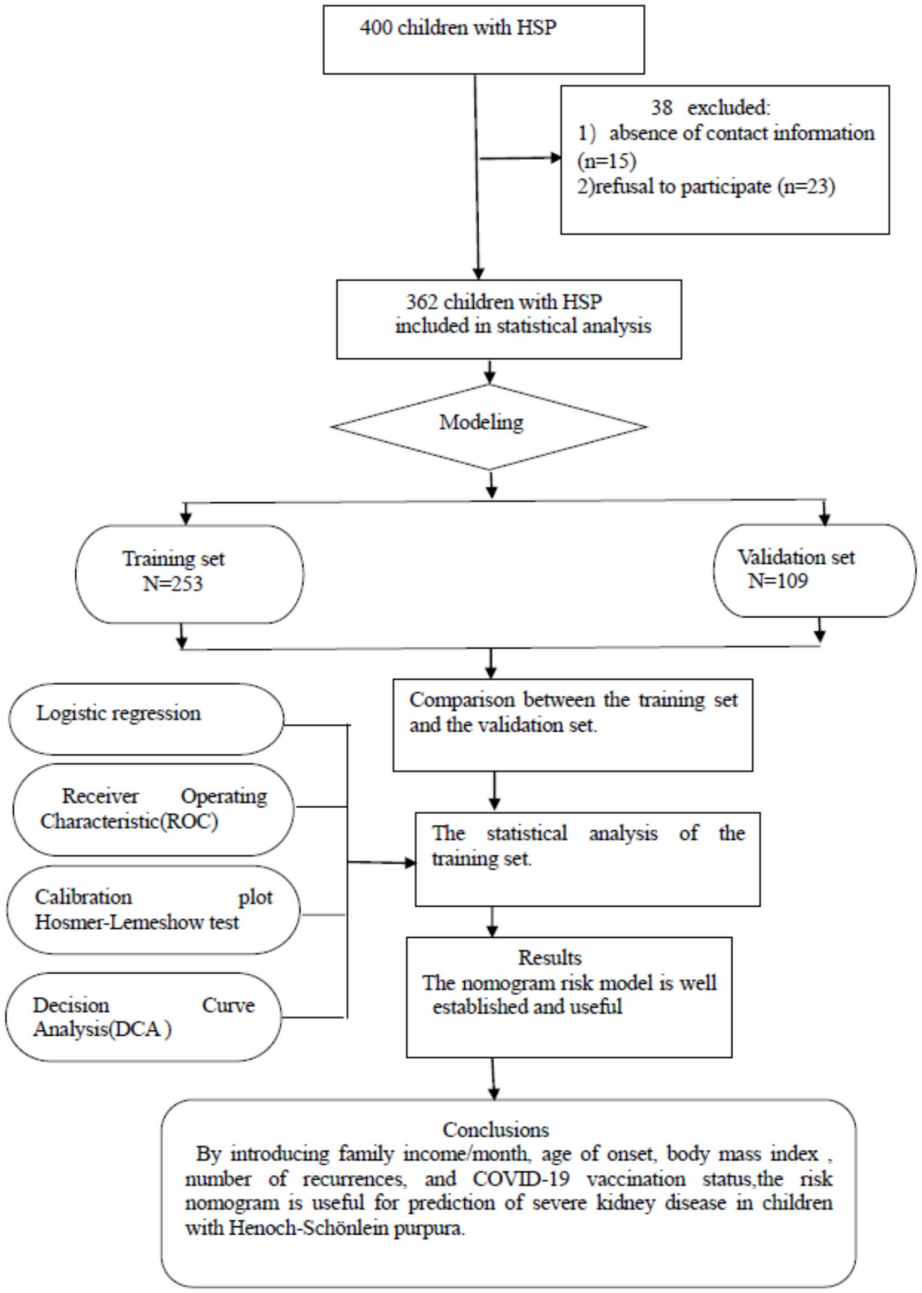
Flowchart of study participant selection. A total of 400 patients with Henoch–Schönlein purpura were enrolled based on the diagnostic criteria for the disease. However, 15 patients could not be contacted due to incorrect phone numbers, and 23 parents refused to participate in the survey.

For each hospitalized patient, data were extracted from both electronic and paper medical records. The study’s data were sourced from the inpatient electronic medical record system at Anhui Provincial Children’s Hospital. Demographic information for all children, including gender, age of onset, BMI, and the number of recurrences, was recorded in their medical records. Parental information and the child’s COVID-19 vaccination history were obtained through questionnaires. Figure 1 illustrated the complete standard technical roadmap.

### 2.2 Survey tools

#### 2.2.1 Questionnaire development

The questionnaire was specifically designed by the research team to collect comprehensive data on demographic details, parental education level, family income, and the child’s COVID-19 vaccination history. The design process involved the following steps:

The content and structure of the questionnaire were informed by a thorough review of previous studies on HSP and associated risk factors.

Pediatric nephrologists were consulted to ensure that the questions were relevant, clear, and scientifically robust.

The initial draft was pre-tested with a small cohort of 20 parents to assess its clarity, relevance, and ease of comprehension. Feedback from the pilot study was used to refine the questionnaire before its final implementation.

#### 2.2.2 Delivery method

The questionnaire was administered using a hybrid approach to maximize response rates. For parents present during their child’s hospitalization, the questionnaires were completed on-site after a detailed explanation of the study’s purpose and objectives by trained researchers. In cases where the parents were not available in person, the research team conducted a telephonic survey to ensure their participation.

#### 2.2.3 Data collection and quality control

This study utilized a convenience sampling approach to select participants based on specific criteria. Their parents of participants were able to take part in the questionnaire survey. Team members received standardized training and thoroughly explained the study’s purpose and procedures to participants before the survey, ensuring that informed consent was properly obtained. During the survey, researchers were on-site to address any questions and ensure that participants fully understood the questionnaire. The collection of questionnaires and data recording were rigorously monitored, with continuous team coordination to maintain study’s accuracy, reliability, and overall quality. This rigorous oversight helped safeguard the integrity of data and ensured that the study adhered to the highest standards of research practice.

#### 2.2.4 Ethical statement

The study was reviewed and approved by the Medical Research Ethics Committee at Anhui Provincial Children’s Hospital (Approval No. EYLL-2023-023), and written informed consent was obtained from parents of all participants. Data were anonymized and de-identified prior to analysis.

### 2.3 Statistical analysis

#### 2.3.1 Statistical methods

All statistical analyses were conducted using SPSS 23.0 (IBM) and R software (version 3.6.1). The Shapiro–Wilk test was employed to evaluate the normality of continuous variables. When data were found to deviate from a normal distribution, the Mann–Whitney *U* test was used instead of the *t*-test, as it is more appropriate for non-normally distributed data. This decision was made because the t-test assumes normality in the data, and the Mann–Whitney *U* test is a non-parametric alternative that does not require such assumptions. Non-normally distributed data were reported as median (interquartile range), while categorical variables were compared using the Chi-square test or Fisher’s exact test, as appropriate. Missing data accounted for less than 5% of the dataset. Missing questionnaire responses were resolved through follow-up phone calls, and clinical data gaps were cross-referenced with hospital records. For variables with less than 2% missing values, imputation was performed using the mean for normally distributed data and the median for non-normally distributed data. Cases with more than 30% missing fields were excluded from the analysis. For variables with 2–30% missing values, sensitivity analyses were conducted to evaluate potential biases and confirm the robustness of results. Factors with a *p*-value <0.05 in univariate analysis were considered for inclusion in multivariate logistic regression to identify independent predictors of HSPN. Only variables that met the assumptions of linearity of log-odds (verified through scatter plots and Box-Tidwell tests) and showed no multicollinearity (Variance Inflation Factor < 10) were included in the final model. A nomogram was developed by converting regression coefficients from the multivariate logistic regression into a 0–100 point scale. Each variable was assigned points proportional to its effect size, and the total score was used to estimate the predicted probability of HSPN. The nomogram’s performance was rigorously evaluated. Discrimination was assessed using the area under the receiver operating characteristic (ROC) curve (AUC). Calibration was evaluated using calibration plots with 1,000 bootstrap resamples and the Hosmer-Lemeshow test. Additionally, decision curve analysis (DCA) was conducted to evaluate the net clinical benefit of the model across a range of threshold probabilities. Statistical significance was set at *p* < 0.05.

#### 2.3.2 Data analysis

##### 2.3.2.1 Training and validation splits

Random assignment was used to place all patients into either the training group (70%, *n* = 253) or the validation group (30%, *n* = 109). Analysis of the initial data showed that there were no notable differences between training group and validation group (*p* > 0.05, Table 1).

**Table 1:** Balance test between the training set and validation set.

Variables	Total (*n* = 362)	Test (*n* = 109)	Train (*n* = 253)	Statistic	*p*
Ageofonset, M (Q₁, Q₃)	7.00 (5.50, 10.00)	8.00 (6.00, 10.00)	7.00 (5.00, 10.00)	Z = –1.36	0.175
BMI, M (Q₁, Q₃)	16.09 (14.72, 18.55)	16.14 (14.93, 19.13)	16.00 (14.61, 18.34)	Z = –0.71	0.479
Renal involvement, *n* (%)				χ^2^ = 0.02	0.881
1	218 (60.22)	65 (59.63)	153 (60.47)		
2	144 (39.78)	44 (40.37)	100 (39.53)		
Number of recurrences, *n* (%)				χ^2^ = 5.78	0.055
1	220 (60.77)	64 (58.72)	156 (61.66)		
2	89 (24.59)	22 (20.18)	67 (26.48)		
3	53 (14.64)	23 (21.10)	30 (11.86)		
Gender, *n* (%)				χ^2^ = 0.68	0.410
1	184 (50.83)	59 (54.13)	125 (49.41)		
2	178 (49.17)	50 (45.87)	128 (50.59)		
Father’s level of education, *n* (%)				χ^2^ = 2.02	0.365
1	170 (46.96)	57 (52.29)	113 (44.66)		
2	94 (25.97)	27 (24.77)	67 (26.48)		
3	98 (27.07)	25 (22.94)	73 (28.85)		
Mother’s level of education, *n* (%)				χ^2^ = 1.71	0.425
1	187 (51.66)	62 (56.88)	125 (49.41)		
2	81 (22.38)	22 (20.18)	59 (23.32)		
3	94 (25.97)	25 (22.94)	69 (27.27)		
Family income/month, *n* (%)				χ^2^ = 1.58	0.665
1	42 (11.60)	12 (11.01)	30 (11.86)		
2	115 (31.77)	30 (27.52)	85 (33.60)		
3	141 (38.95)	46 (42.20)	95 (37.55)		
4	64 (17.68)	21 (19.27)	43 (17.00)		
COVID-19 vaccination, *n* (%)				χ^2^ = 0.03	0.853
1	28 (7.73)	8 (7.34)	20 (7.91)		
2	334 (92.27)	101 (92.66)	233 (92.09)		

Z, Mann–Whitney test; χ^2^, Chi-square test; M, median; Q₁, 1st Quartile; Q₃, 3st quartile. *p* < 0.05 suggested statistical significance.

##### 2.3.2.2 Statistical analysis of the training set

In training group (*n* = 253), baseline characteristics were divided and analyzed based on the presence of renal involvement (Table 2). The study found that parental education level, family income/month, BMI, age of onset, number of recurrences, and COVID-19 vaccination status were significant risk factors for development of nephritis in patients with HSP (*p* < 0.05).

**Table 2:** Basic characteristics and analysis of differences.

Variables	Total (*n* = 253)	1 (*n* = 153)	2 (*n* = 100)	Statistic	*p*
Age of onset, M (Q₁, Q₃)	7.00 (5.00, 10.00)	6.50 (5.00, 8.00)	9.00 (6.00, 11.00)	Z = -4.03	**<0.001**
BMI, M (Q₁, Q₃)	16.00 (14.61, 18.34)	15.39 (14.40, 17.54)	16.95 (15.04, 20.00)	Z = -3.78	**<0.001**
Number of recurrences, *n* (%)				χ^2^ = 42.11	**<0.001**
One	156 (61.66)	118 (77.12)	38 (38.00)		
Two	67 (26.48)	28 (18.30)	39 (39.00)		
Three or more recurrences	30 (11.86)	7 (4.58)	23 (23.00)		
Gender, *n* (%)				χ^2^ = 0.38	0.536
Female	125 (49.41)	78 (50.98)	47 (47.00)		
Male	128 (50.59)	75 (49.02)	53 (53.00)		
Father’s level of education, *n* (%)				χ^2^ = 6.32	**0.042**
College or above	113 (44.66)	63 (41.18)	50 (50.00)		
High school	67 (26.48)	37 (24.18)	30 (30.00)		
Lower than high school	73 (28.85)	53 (34.64)	20 (20.00)		
Mother’s level of education, *n* (%)				χ^2^ = 10.98	**0.004**
College or above	125 (49.41)	66 (43.14)	59 (59.00)		
High school	59 (23.32)	34 (22.22)	25 (25.00)		
Lower than high school	69 (27.27)	53 (34.64)	16 (16.00)		
Family income/month, *n* (%)				χ^2^ = 24.81	**<0.001**
> 20,000 RMB	30 (11.86)	28 (18.30)	2 (2.00)		
10,000–20,000 RMB	85 (33.60)	57 (37.25)	28 (28.00)		
5,000–10,000 RMB	95 (37.55)	51 (33.33)	44 (44.00)		
< 5,000 RMB	43 (17.00)	17 (11.11)	26 (26.00)		
COVID-19 vaccination, *n* (%)				χ^2^ = 5.90	**0.015**
Yes	20 (7.91)	7 (4.58)	13 (13.00)		
No	233 (92.09)	146 (95.42)	87 (87.00)		

Z, Mann–Whitney test; χ^2^, Chi-square test; M, median, Q₁, 1st quartile; Q₃, 3st quartile. *p* < 0.05 suggested statistical significance.

**Figure 2:**
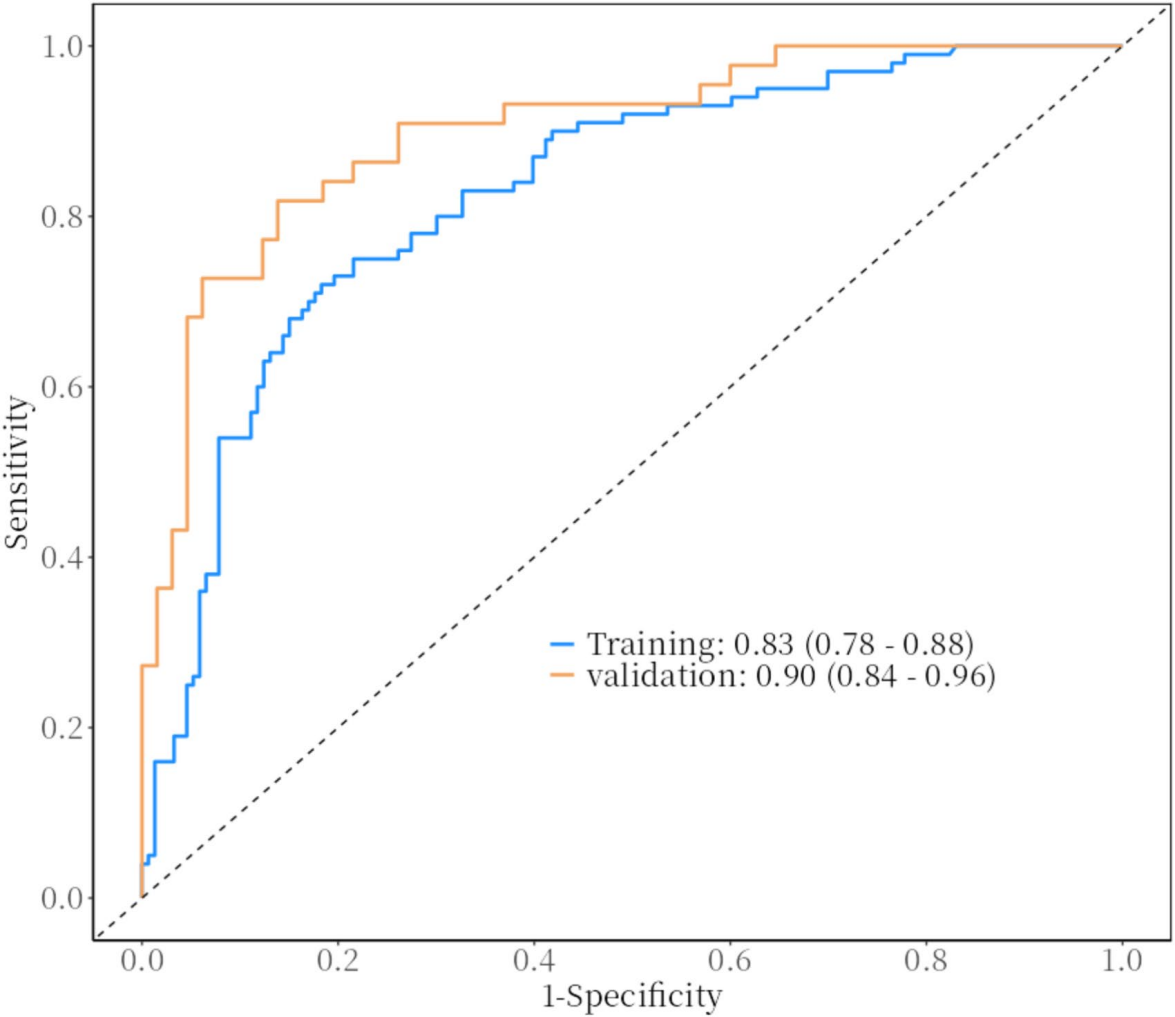
ROC curve and AUC.

**Figure 3:**
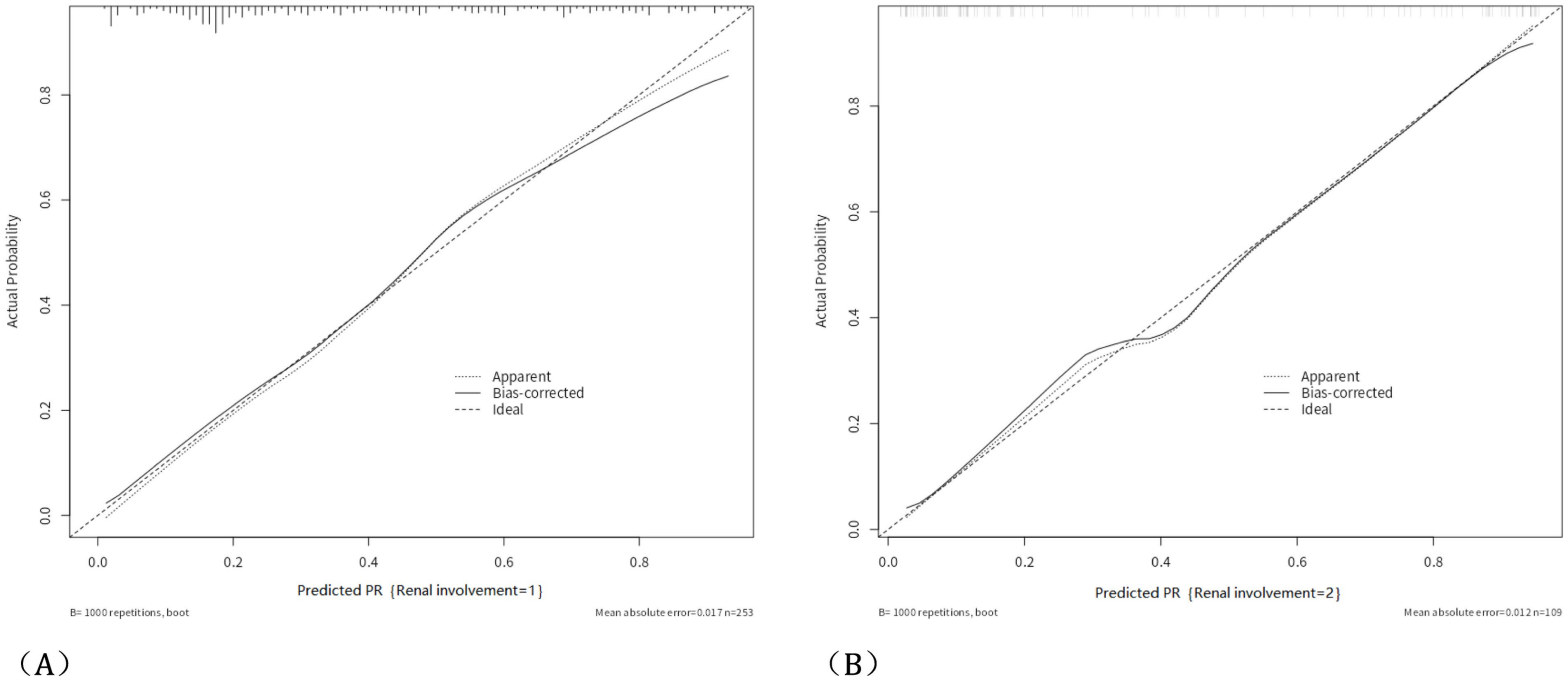
Calibration curve and HL analysis. The calibration curve of the nomogram predicting HSPN in the training group, with a Hosmer-Lemeshow test *p*-value of 0.562 in the training group **(A)**; and the calibration curve of the nomogram predicting HSPN in the validation group, with a Hosmer-Lemeshow test *p*-value of 0.332 in the validation group **(B)**.

Tables 3, 4 univariate and multivariate logistic regression analyses, using generalized estimating equations, were performed to analyze the factors influencing HSPN (Tables 3, 4). The results are expressed as *β* coefficients and odds ratios (OR) with corresponding 95% confidence intervals (95% CI). The analysis included seven variables from the previous step (2), revealing that family income per month (OR, 11.25; 95% CI: 2.17–58.37; *p* = 0.004 for 10,000–20,000 RMB; OR, 12.73; 95% CI: 2.51–64.63; *p* = 0.002 for 5,000–10,000 RMB; OR, 19.32; 95% CI: 3.28–113.95; *p* = 0.001 for less than 5,000 RMB), age at onset (OR, 1.17; 95% CI: 1.03–1.33; *p* = 0.015), two recurrences (OR, 4.80; 95% CI: 2.37–9.69; *p* < 0.001), three or more recurrences (OR, 11.99; 95% CI: 3.93–36.56; *p* < 0.001), BMI (OR, 1.12; 95% CI: 1.01–1.25; *p* = 0.047), and COVID–19 vaccination status (OR, 0.26; 95% CI: 0.08–0.82; *p* = 0.022) were identified as independent risk factors for the development of nephritis in patients with HSP (Figures 2, 3).

**Table 3:** Results of univariate logistic regression.

Variables	β	S.E	Z	*P*	OR (95%CI)
Number of recurrences					
One					1.00 (Reference)
Two	1.46	0.31	4.72	**<0.001**	4.33 (2.36 ~ 7.94)
Three or more recurrences	2.32	0.47	4.94	**<0.001**	10.20 (4.06 ~ 25.64)
Gender					
Female					1.00 (Reference)
Male	0.16	0.26	0.62	0.536	1.17 (0.71 ~ 1.94)
Father’s level of education					
College or above					1.00 (Reference)
High school	0.02	0.31	0.07	0.945	1.02 (0.56 ~ 1.88)
Lower than high school	−0.74	0.32	−2.30	**0.022**	0.48 (0.25 ~ 0.90)
Mother’s level of education					
College or above					1.00 (Reference)
High school	−0.20	0.32	−0.61	0.540	0.82 (0.44 ~ 1.54)
Lower than high school	−1.09	0.34	−3.22	**0.001**	0.34 (0.17 ~ 0.65)
Family income/month					
> 20,000 RMB					1.00 (Reference)
10,000–20,000 RMB	1.93	0.77	2.51	**0.012**	6.88 (1.53 ~ 30.95)
5,000–10,000 RMB	2.49	0.76	3.28	**0.001**	12.08 (2.72 ~ 53.60)
< 5,000 RMB	3.06	0.80	3.85	**<0.001**	21.41 (4.50 ~ 101.83)
COVID-19 vaccination					
No					1.00 (Reference)
Yes	−1.14	0.49	−2.33	**0.020**	0.32 (0.12 ~ 0.84)
Age of onset	0.19	0.05	4.03	**<0.001**	1.20 (1.10 ~ 1.32)
BMI	0.16	0.04	3.90	**<0.001**	1.17 (1.08 ~ 1.27)

OR, odds ratio; CI, confidence interval.

**Table 4:** Results of multivariate logistic regression.

Variables	*β*	S.E	*Z*	*P*	OR (95%CI)
Intercept	−5.72	1.43	−4.00	**<0.001**	0.00 (0.00 ~ 0.05)
Number of recurrences					
One					1.00 (Reference)
Two	1.57	0.36	4.37	**<0.001**	4.80 (2.37 ~ 9.69)
Three or more recurrences	2.48	0.57	4.37	**<0.001**	11.99 (3.93 ~ 36.56)
Gender					
Female					1.00 (Reference)
Male	0.05	0.33	0.16	0.876	1.05 (0.55 ~ 2.00)
Father’s level of education					
College or above					1.00 (Reference)
High school	0.80	0.46	1.75	0.079	2.23 (0.91 ~ 5.47)
Lower than high school	0.88	0.54	1.62	0.105	2.40 (0.83 ~ 6.91)
Mother’s level of education					
College or above					1.00 (Reference)
High school	0.15	0.44	0.35	0.729	1.17 (0.49 ~ 2.77)
Lower than high school	−1.01	0.56	−1.82	0.069	0.36 (0.12 ~ 1.08)
Family income/month					
> 20,000 RMB					1.00 (Reference)
10,000–20,000 RMB	2.42	0.84	2.88	**0.004**	11.25 (2.17 ~ 58.37)
5,000–10,000 RMB	2.54	0.83	3.07	**0.002**	12.73 (2.51 ~ 64.63)
< 5,000 RMB	2.96	0.91	3.27	**0.001**	19.32 (3.28 ~ 113.95)
COVID-19vaccination					
No					1.00 (Reference)
Yes	−1.33	0.58	−2.29	**0.022**	0.26 (0.08 ~ 0.82)
Age of onset	0.16	0.06	2.42	**0.015**	1.17 (1.03 ~ 1.33)
BMI	0.11	0.06	1.98	**0.047**	1.12 (1.01 ~ 1.25)

OR, odds ratio; CI, confidence interval.

The ROC curves of HSPN in the training and validation groups: The ROC curve based on the nomogram of the training group has an AUC of 0.83; the ROC curve from the validation group has an AUC of 0.90. The figure shows the estimated values of AUC and their 95%.

**Figure 4:**
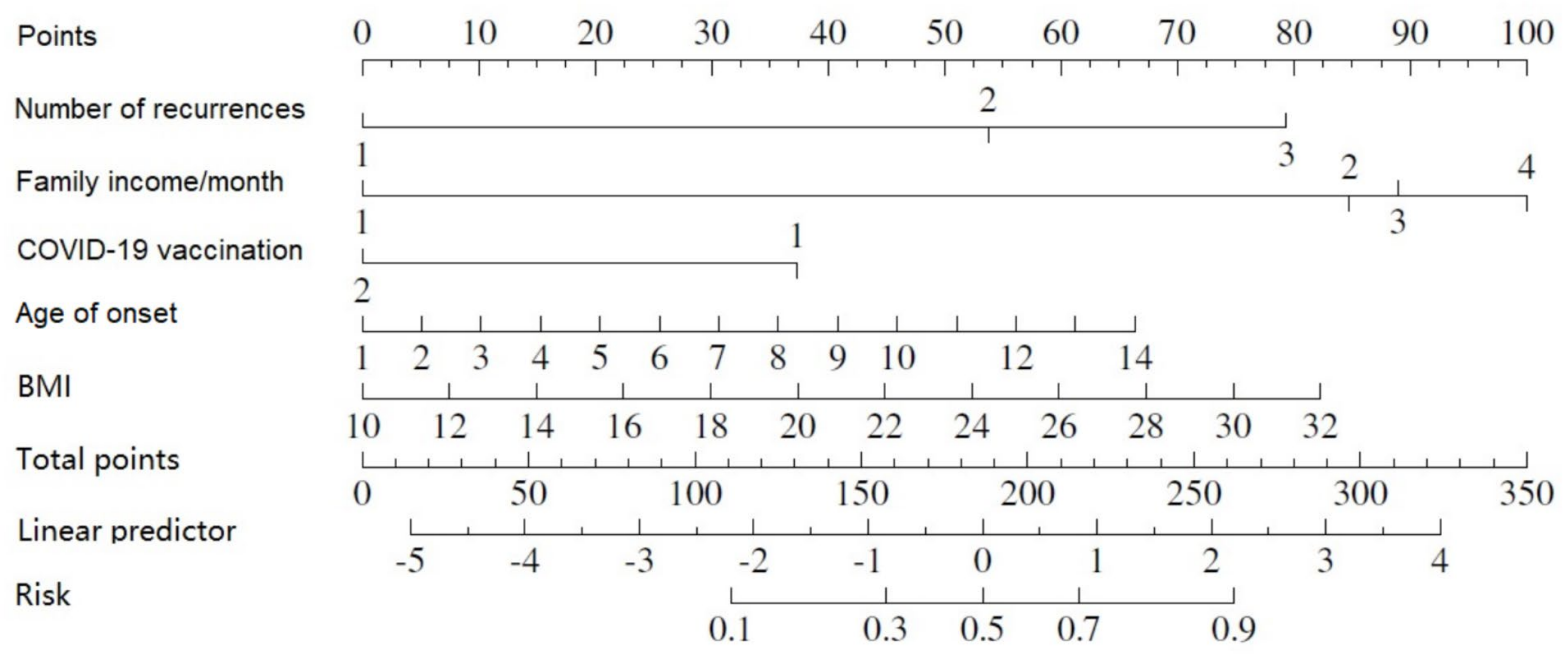
The predictive performance of the nomogram for the prevalence of HSPN.

As indicated by the outcomes of the binary logistic regression analysis, five variables were incorporated into the nomogram (Figure 4). With the nomogram, a specific score is designated for each variable value, and the sum of these scores can be aligned vertically to estimate the probability of developing nephritis in patients with HSP.A higher total score indicates an enhanced risk of contracting HSPN (Table 5).

**Table 5:** Results of Training and Validation Analysis.

Data	AUC (95%CI)	Accuracy (95%CI)	Sensitivity (95%CI)	Specificity (95%CI)	PPV (95%CI)	NPV (95%CI)	Cut off
Train	0.83 (0.78–0.88)	0.78 (0.72–0.83)	0.82 (0.76–0.88)	0.72 (0.63–0.81)	0.82 (0.76–0.88)	0.72 (0.63–0.81)	0.449
Test	0.90 (0.84–0.96)	0.82 (0.73–0.88)	0.82 (0.72–0.91)	0.82 (0.70–0.93)	0.87 (0.78–0.95)	0.75 (0.63–0.87)	0.449

DCA demonstrates that the nomogram provides significant clinical benefit across threshold probabilities, outperforming default strategies by effectively balancing true positive predictions and avoiding unnecessary treatments. This figure shows the DCA curve for the training set (which shows better net benefit of the model within the range of 0.1–0.87) and the DCA curve for the validation set (which shows better net benefit of the model within the range of 0.1–0.98).

## 3 Results

### 3.1 Participant characteristics

This study analyzed data from 362 participants, who were randomly allocated into a training group (70%, *n* = 253) and a validation group (30%, *n* = 109). No notable differences were observed between the training and validation groups in baseline comparisons (*p* > 0.05, Tables 1, 6).

**Table 6:** Independent risk factors for nephritis in children with HSP.

Variables	Odds ratio (OR)	95% Confidence interval (CI)	*p-*value
Family income per month: ¥10,000–20,000	11.25	2.17–58.37	0.004
Family income per month: ¥5,000–10,000	12.73	2.51–64.63	0.002
Family income per month: < ¥5,000	19.32	3.28–113.95	0.001
Age of onset	1.17	1.03–1.33	0.015
Two recurrences	4.80	2.37–9.69	<0.001
Three or more recurrences	11.99	3.93–36.56	<0.001
BMI	1.12	1.01–1.25	0.047
COVID-19 vaccination	0.26	0.08–0.82	0.022

### 3.2 Independent risk factors for nephritis

#### 3.2.1 Nomogram performance and DCA

Built on the results from logistic regression, a nomogram was developed to predict the likelihood of HSPN using factors such as family income, age of onset, number of recurrences, BMI, and COVID-19 vaccination status. Each factor was assigned a score, and the total score was used to estimate the probability of developing nephritis. The nomogram demonstrated strong predictive performance. The ROC curve revealed an AUC of 0.83 for the training group and 0.90 for the validation group. Sensitivity was 82% in both groups, while specificity was 72% in the training group and 82% in the validation group. Figures 2^5^ illustrated the ROC curve and DCA results, respectively, showing consistent performance across both groups.

**Figure 5:**
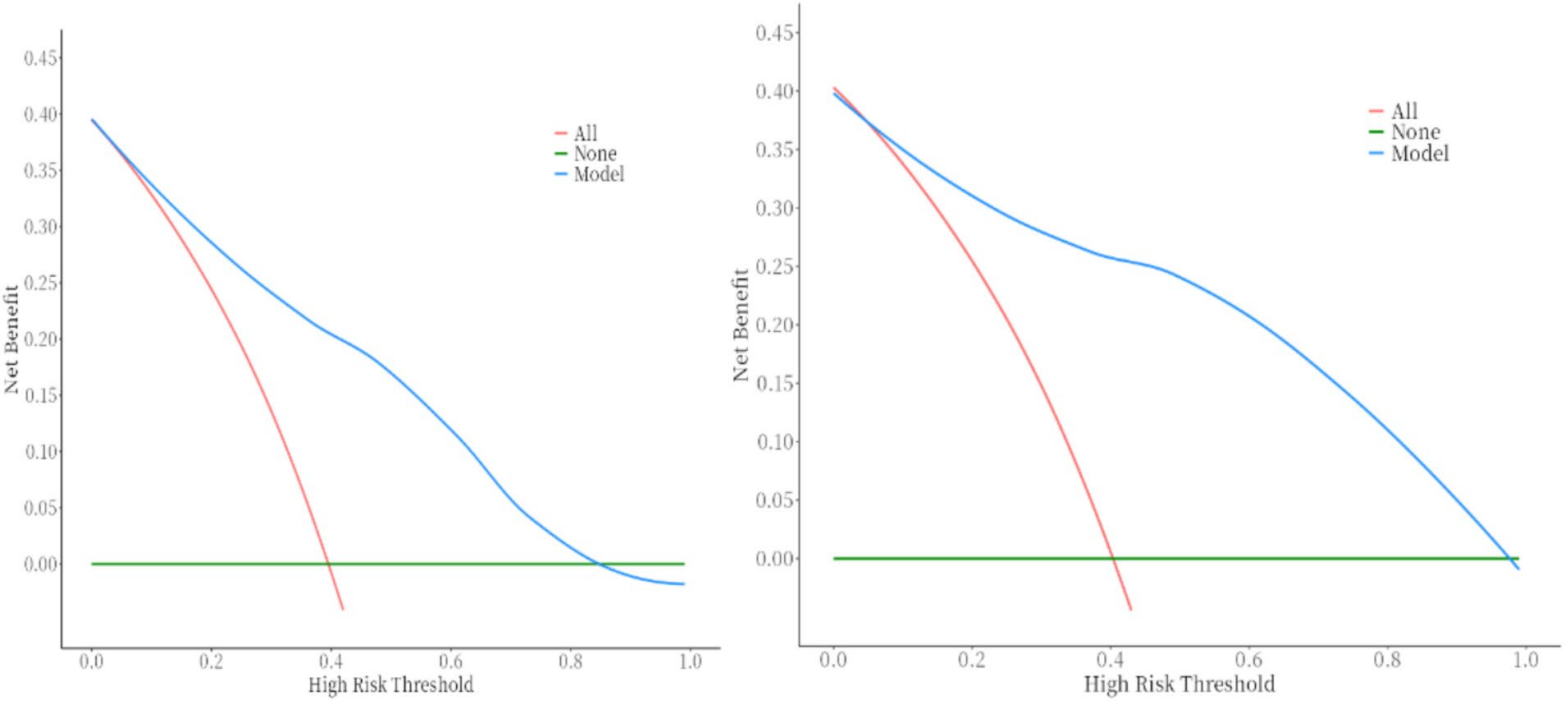
DCA curve.

## 4 Discussion

IgAVN is a prevalent secondary glomerular disease in children and a major cause of end-stage kidney disease (ESKD) (15). This chronic condition is difficult to treat and significantly affects the quality of life of affected children, highlighting the need for proactive prevention. In this study, a nomogram was developed to predict the risk of nephritis in children with HSP. The analysis revealed that family income/month, age of onset, number of recurrences, BMI, and COVID-19 vaccination status were associated with a heightened likelihood of developing nephritis. Compared to other predictive models for HSPN, our nomogram offers several advantages, particularly its inclusion of family environmental factors and COVID-19 vaccination status, which have not been addressed in other studies. The nomogram demonstrated strong discriminative ability, effectively distinguishing nephritis from non-nephritis patients in both the training and validation groups, with AUC values of 0.83 and 0.90, respectively. Additionally, DCA showed that it offers significant clinical net benefits. This tool can be integrated into clinical practice to estimate the probability of nephritis in children with HSP.

Among the three general traits examined—BMI, age of onset, and number of recurrences—BMI emerged as a significant risk factor for HSPN. BMI, calculated by dividing weight (kg) by height squared (m^2^), serves as a gauge for overweight status. Many studies have shown that obesity is a major factor in kidney disease. For example, the Framingham study (16) found that a one-unit increase in BMI over 20 years correlates with a 20% higher risk of developing kidney disease. Literature (17, 18) also indicates that obesity in children with HSP raises the risk of kidney damage and severe renal involvement, and serves as a predictor of progression to ESKD. Our study confirms that as BMI increases, so does the risk of renal involvement in HSP patients.

Several studies have demonstrated that the age of onset is a risk factor for renal involvement in children with HSP. Research in Chinese children by Zhao (17) found that an onset age older than 6 years is a significant risk factor for renal involvement. Similarly, Japanese research by Sano (19) and Jauhola (20) identified ages over 4 and 8 years as risk factors, respectively. A meta-analysis (21) confirmed that older children face a greater risk of renal involvement. Our research also shows that a later age of onset correlates with a higher risk of renal involvement.

Research by Outi Jauhola (22) and others indicates that patients with nephritis (OR 3.7) and those older than 8 years (OR 4.6) have higher recurrence rates. Wei-Te Lei’s study (23) found that the risk of recurrence is greater in patients with renal involvement. Our study confirms that the likelihood of kidney involvement in HSP increases with the number of recurrences.

In comparison with previous studies, our data align with the findings of Ke Wang (24), who also did not identify gender as a risk factor for renal involvement.

Our study suggests that low family income is an independent risk factor for renal involvement in children. This may be related to challenges faced by low-income families in accessing medical resources, ensuring proper nutrition, and securing mental health care, among other factors. Given limited research on the impact of household economic status on kidney health, pediatricians should focus on children from low-income backgrounds. Addressing these risk factors promptly is crucial for improving health outcomes and reducing disparities in care.

With the rapid spread of COVID-19, vaccination has become essential in combating the pandemic. Some studies suggest that COVID-19 vaccination may induce HSP (9) and trigger recurrences of kidney diseases (25). Increasing evidence shows that organs such as the kidneys are affected due to widespread ACE2 receptors. COVID-19 vaccination may provoke autoimmune diseases, including autoimmune glomerulonephritis (26). Reports suggest that vaccination might precipitate IgA nephropathy (27) and lead to relapses in previously treated cases (28). The potential mechanisms of kidney injury related to COVID-19 vaccination include SARS-CoV-2 infecting renal cells, as supported by histopathological findings (29, 30), and lymphocytic endotheliitis in the kidneys, leading to microvascular dysfunction (31). Our study indicates that COVID-19 vaccination is an independent risk factor for kidney involvement in children with HSP. Although emerging evidence suggests a link between vaccination and autoimmune conditions like HSP, the observational nature of this study does not allow us to definitively confirm causality. Other factors, such as prior infections, genetic predisposition, or concurrent treatments, may also contribute. While vaccination carries some risk of triggering autoimmune responses, its overwhelming benefits in preventing severe illness, hospitalization, and death, especially in vulnerable populations, should be recognized. Given the rapid spread of COVID-19, the protective benefits outweigh the rare risk of adverse events like HSP. Further research is needed to explore the potential link between vaccination and nephritis in children with HSP.

In conclusion, low family income, age of onset, BMI, number of recurrences, and COVID-19 vaccination are independent risk factors for HSP nephritis in children in the Anhui region. This study enhances our understanding of familial risk factors for HSPN and the potential impact of COVID-19 vaccination. Additionally, our nomogram offers a cost-effective and widely applicable tool for predicting the likelihood of HSPN. These conclusions provide valuable insights for future clinical and public health practices, guiding evidence-based strategies to optimize health outcomes.

The study has several limitations: Retrospective Design: The study’s retrospective nature limits causality. Single-Center Bias: Conducted at one institution, which may introduce selection and information biases. The sample may not fully represent the broader pediatric population with HSP in other regions. Selection Bias: Convenience sampling from a specific hospital in Anhui Province limits generalizability. Incomplete Data: Missing data may introduce biases despite efforts to minimize it. Recall Bias: Self-reported data from parents may introduce inaccuracies, especially regarding vaccination history and socioeconomic status. Given these limitations, the results should be interpreted with caution. Future multi-center, prospective cohort studies with robust data collection methods are needed to validate these findings and address potential biases.

## Data Availability

The original contributions presented in the study are included in the article/supplementary material, further inquiries can be directed to the corresponding author/s.
